# Inhibition of estrogen biosynthesis enhances lymphoma growth in mice

**DOI:** 10.18632/oncotarget.7843

**Published:** 2016-03-02

**Authors:** Gergely Talaber, Konstantin Yakimchuk, Jiyu Guan, Jose Inzunza, Sam Okret

**Affiliations:** ^1^ Department of Biosciences and Nutrition, Karolinska Institutet, NOVUM, Huddinge, Sweden

**Keywords:** lymphoma, sex hormones, androgens, aromatase inhibitor, gender difference

## Abstract

Most lymphomas show higher incidence and poorer prognosis in males compared to females. However, the endocrine contribution to this gender difference is not entirely known. Here we show that castration accelerates lymphoma growth in C57BL6 male mice grafted with murine EG7 T cell lymphoma cells. However, the androgen receptor antagonist Bicalutamide did not affect lymphoma growth, suggesting no impact of androgen receptor signaling on lymphoma progression. In contrast, inhibition of androgen-to-estrogen conversion by the aromatase inhibitor (AI) Letrozole induced faster lymphoma growth in mice, suggesting that androgens impact lymphoma growth through its conversion to estrogens. This was supported by the inability of dihydrotestosterone, which is not converted to estrogens by aromatase, to influence lymphoma growth in castrated male mice. Lymphoma growth was also stimulated in immunocompromised mice grafted with human B cell lymphoma (Granta-519) and treated with either reversible or irreversible AIs, showing that the blockage of estrogen synthesis caused enhanced growth of both murine T and human B cell lymphomas and with different AIs. Additionally, AI-treated EG7 lymphomas showed accelerated growth not only in male but also in intact female mice. Altogether, our results demonstrate that aromatase inhibition accelerates lymphoma growth but not androgens *per se*, highlighting a protective role of estrogens in lymphoma pathogenesis. These results also raise concern that the use of AIs in women with breast cancer might enhance lymphoma progression.

## INTRODUCTION

Most lymphomas, particularly Non-Hodgkin lymphomas (NHL), show a higher incidence and poorer prognosis in males than in females [1, 2]. As an explanation behind this gender difference, high estrogen levels have been suggested to protect against lymphomas in women, particularly at reproductive age [3]. For example, in Diffuse Large B Cell Lymphoma, the most common lymphoma type, a gender difference is seen in incidence between pre-menopausal women and men but not between post-menopausal women and men [2, 4, 5]. Furthermore, a decreased incidence with increased number of pregnancies has been demonstrated [6, 7] and in several studies, but not all, a lower lymphoma incidence seem to correlate to the use of hormone replacement therapy [3, 8, 9]. Thus, several epidemiological data suggest that NHL may be estrogen-regulated.

Estrogen action in their target tissue is largely mediated through two nuclear receptors, namely estrogen receptor α (ERα) and ERβ [10, 11]. The ERs are widely expressed in many tissues, although ERα and ERβ show different tissue distribution [12–14]. The main ER expressed in both normal lymphocytes as well in lymphoma cells is ERβ while ERα levels are very low or undetectable [14–16].

The major estrogen, estradiol, binds to both ERα and ERβ with similar affinity and is able to act as a potent agonist on both receptors [17]. ERα mainly has a stimulatory effect on cell proliferation and has been shown to be a tumor promoter in, for example, breast cancer, whereas ERβ mainly acts in the opposite way by exerting anti-proliferative and pro-apoptotic effects on multiple healthy [18–22] and tumor cell types, such as in breast [23], prostate [24] and ovarian cancer [25]. We have previously demonstrated this also to be the case for several subtypes of lymphomas [16, 26]. Furthermore, we have also shown that grafted lymphomas grow faster in male mice than in female mice and that this gender difference disappeared in ovariectomized female mice [26]. Furthermore, ERβ agonists were successfully utilized to slow lymphoma growth and inhibiting lymphoma vascularization and dissemination [16].

Although the above studies show a role for estrogens on NHL progression, the role of male sex hormones (androgens) on lymphoma development is uncertain. This is despite the demonstration that androgen receptor (AR) is expressed in at least some lymphoma cells [27]. Furthermore, androgens can exert their action in two ways, i) by a direct effect via binding to the AR or ii) through conversion of androgens to estrogens by the aromatase enzyme [28]. This androgen-to-estrogen conversion by aromatase occurs both in males and females, mainly in the gonads, but also in peripheral tissues such as adipose and brain [29]. Aromatase-mediated androgen-to-estrogen conversion is the source of estrogens in males [30]. Likewise, the main estrogen source in postmenopausal women is the aromatase-mediated conversion in peripheral organs of adrenal-derived androgens [30].

In this study we investigated the effect of androgens on lymphoma growth and their mechanism of action. Since androgens can be converted to estrogens by aromatase, we particularly studied whether androgens had a direct effect on lymphoma growth or if it was mediated through conversion to estrogens.

## RESULTS

### Surgical castration promotes lymphoma growth in male mice

**Figure 1:**
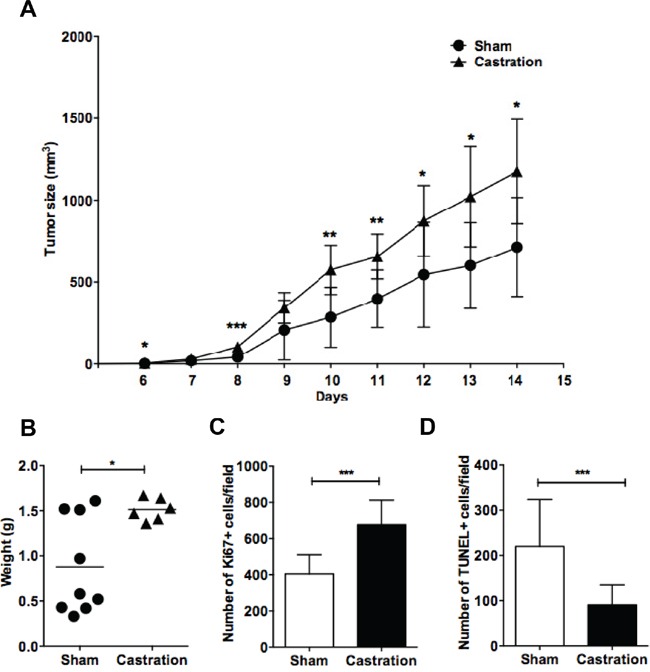
Removal of sex hormones by surgical castration promotes lymphoma growth in male mice Mice were surgically castrated and grafted with EG7 lymphoma cells. **A.** Growth curves show EG7 lymphoma growth in sham-operated (●) (n=9) *vs.* castrated mice (▲) (n=6). **B.** EG7 tumor weights of sham-operated (●) *vs.* castrated (▲) mice at the last experimental day. Each point represents a mouse. **C.** Ki67 positive cells and **D.** TUNEL-positive cells of EG7 lymphoma tumors were counted by immunofluorescent microscopy. *, p<0.05; **, p<0.01, ***p<0.001. Data analyzed by 2-tailed Student t-test.

Previous results have shown that grafted EG7 lymphoma grows faster in male vs. female mice. This difference was attributed to female sex hormones as it was abolished following ovariectomy [26]. Furthermore, estrogen administration inhibited lymphoma growth [16, 26]. However, a possible impact of also male sex hormones on lymphoma growth has not been elucidated. We therefore investigated whether removal of male sex hormones influenced lymphoma growth. Male C57BL6 mice were surgically castrated or sham-operated prior to grafting with EG7 murine T lymphoma cells. Compared to sham-operated mice, EG7 murine T cell lymphoma in castrated mice showed an accelerated growth (Figure 1A). At the end of the experiment, tumors removed from castrated mice had a significantly larger weight compared to sham-operated tumors (Figure 1B) and exhibited a higher number of proliferating tumor cells as assessed by Ki67 staining (Figure 1C). Furthermore, a significantly lower number of apoptotic cells, as determined by TUNEL-staining, were observed (Figure 1D). This suggests that castration and the removal of male sex hormones, similarly to the removal of female sex hormones by ovariectomy [26] induces lymphoma cell proliferation and inhibits apoptosis leading to an overall accelerated lymphoma growth.

### Inhibition of aromatase-mediated androgen to estrogen conversion rather than inhibition of androgen action accelerates lymphoma growth

**Figure 2:**
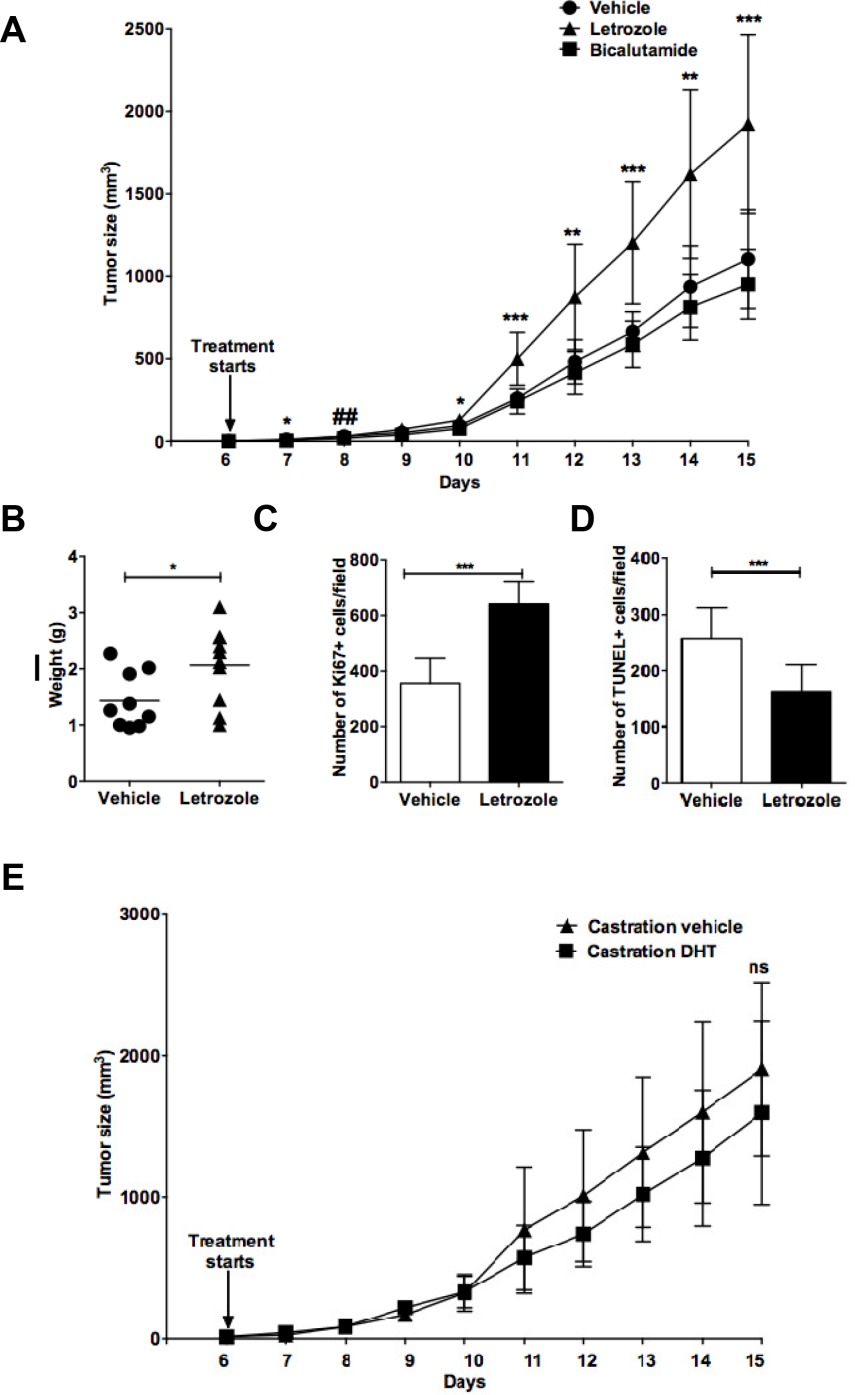
Aromatase inhibition, but not androgen receptor blockade promotes EG7 lymphoma growth in C57BL6 male mice Intact C57BL6 male mice were grafted with EG7 lymphoma cells and treated with the aromatase inhibitor Letrozole (▲) (n=10), the androgen receptor antagonist Bicalutamide (■) (n=9) or vehicle (●) (n=9) starting at the time of development of palpable tumors. **A.** Growth curves of EG7 lymphomas in vehicle *vs.* Letrozole or Bicalutamide-treated animals. **B.** EG7 tumor weights of vehicle *vs.* Letrozole treated mice at the last experimental day. Each point represents a mouse. **C.** Ki67 positive cells and **D.** TUNEL positive cells of EG7 lymphoma tumors from vehicle and Letrozole-treated animals were counted using immunofluorescent microscopy. Vehicle *vs.* Letrozole: *, p<0.05; **, p<0.01, ***, p<0.001; Vehicle *vs.* Bicalutamide:##, p<0.01 (a significant difference was only seen on day 8 (A) but no significant difference was found on any other time-point. No significant difference was seen between vehicle *vs*. Bicalutamide-treated mice with regard to weight at last experimental day and Ki67 or TUNEL positive cells). **E.** Surgically castrated C57BL6 mice were grafted with EG7 lymphoma cells and treated with vehicle (▲) (n=5) or DHT (■) (n=7) daily. Tumor size was measured daily and growth curves are shown. No significant difference was observed within the two groups at any given time-point. Ns= not significant. Data analyzed by 2-tailed Student t-test.

Although castration accelerated tumor growth, it is not possible to conclude that the effect is mediated by loss of androgens or is due to loss of estrogens as estrogens are formed following conversion of androgens to estrogens by the enzyme aromatase [28]. To investigate whether the accelerated lymphoma growth following castration was due to the lack of androgens acting on AR or due to a lack of conversion of androgens to estrogens, we treated intact C57BL6 male mice grafted with EG7 lymphoma cells with the AI Letrozole or the androgen receptor (AR) antagonist Bicalutamide. As can be seen from Figure 2A, aromatase inhibition by Letrozole but not AR antagonism by Bicalutamide significantly accelerated lymphoma growth. Similar to the castration experiment (Figure 1 above), lymphomas removed from Letrozole-treated mice had a significantly higher weight compared to vehicle-treated mice (Figure 2B), more proliferating (Ki67-positive) cells (Figure 2C) and less apoptotic tumor cells (Figure 2D). This suggests that rather estrogens formed from androgens by aromatization than androgens acting via the AR influence lymphoma progression.

To further confirm that androgen-to-estrogen conversion was the cause affecting lymphoma growth, surgically castrated male C57BL6 mice grafted with EG7 lymphoma cells were treated with dihydrotestosterone (DHT), which like testosterone is an AR agonist but in contrast to testosterone cannot be converted to estrogens by aromatase [31]. As shown in Figure 2E, DHT treatment of castrated mice with EG7 lymphomas did not significantly influence tumor growth, further ruling out a direct role of androgens on lymphoma growth (no significance detected at any of the time points).

### Growth of also human B cell lymphoma (Granta-519) grafted to immunodeficient mice is enhanced by different AIs

**Figure 3:**
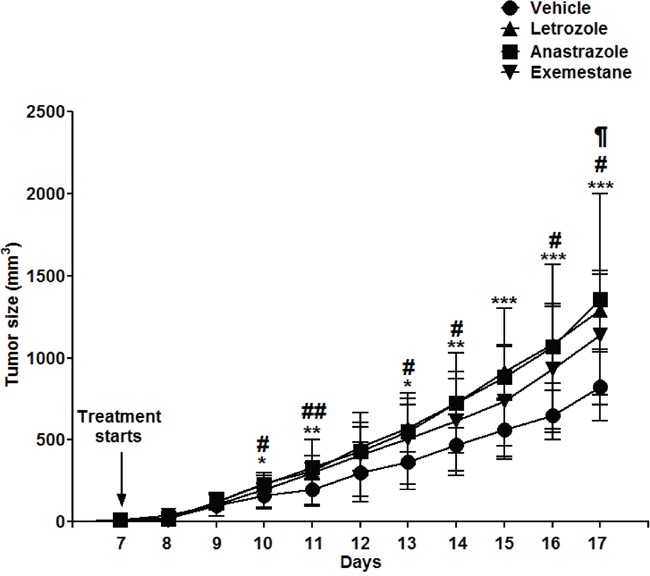
Several different aromatase inhibitors promote lymphoma growth in male NSG mice grafted with human mantle B cell lymphomas cells (Granta-519) Intact male NSG mice were grafted with human Granta-519 Mantle cell lymphoma cells and treated with different aromatase inhibitors, Letrozole (▲) (n=8), Anastrazole (■) (n=9), Exemestane (▼) (n=9) or vehicle (●) (n=9) from the time of development of palpable tumors. Tumor growth curves are shown.* p<0.05, ** p<0.01, ***p<0.001 vehicle *vs.* Letrozole, # p<0.05, ## p<0.01 vehicle *vs*. Anastrazole, ¶ p<0.05 vehicle *vs.* Exemestane. No significant difference in lymphoma tumor growth was seen between any of the aromatase inhibitors. Data analyzed by 2-tailed Student t-test.

To demonstrate that aromatase inhibition indeed stimulates lymphoma growth and that it is not specific for Letrozole, or that the effect on lymphoma growth is not restricted to the murine T cell lymphoma EG7 but also affects human B cell lymphoma and, finally, is not dependent on an intact immune system, we grafted immune deficient NSG mice with human lymphoma B cells (Granta-519). Aromatase inhibition by Letrozole or by other AIs, Anastrazole and Exemestane, the latter being a steroidal irreversible inhibitor [32], enhanced lymphoma growth similar to the results seen in intact C57BL6 mice using a murine T cell lymphoma (Figure 3). Thus, the results showed that several AIs acting through slightly different mechanisms stimulated lymphoma growth, strongly suggesting that the lymphoma promoting effect is mediated through inhibition of estrogen synthesis by inhibition of aromatase. Furthermore, the results demonstrated that the effect is not restricted to a murine T cell lymphoma but also affected human B cell lymphoma. Finally, the experiment showed that the effect is independent of an intact immune system.

### Aromatase inhibition induces accelerated lymphoma growth in intact female mice

**Figure 4:**
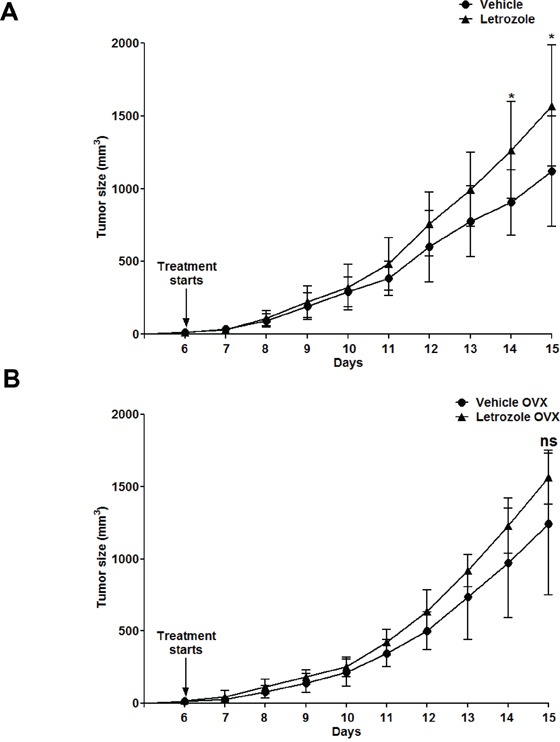
Aromatase inhibition with Letrozole accelerates lymphoma growth in intact but not in ovariectomized female C57BL6 mice C57BL6 female mice were grafted with EG7 cells. **A.** Growth curves of Vehicle (●) (n=10) and Letrozole (▲) (n=8) treated non-operated C57BL6 female mice. * p<0.05 Vehicle *vs.* Letrozole. **B.** Growth curves of ovariectomized (OVX) mice treated with Vehicle (●) (n=10) and Letrozole (▲) (n=7). No significant difference was observed within the two groups at any given time-point. Ns, not significant. Data analyzed by 2-tailed Student t-test.

Aromatase inhibition is widely used as adjuvant therapy in female patients with hormone-sensitive breast cancer [33]. We therefore asked whether inhibition of aromatase-mediated androgen-to-estrogen conversion could have the same stimulatory effect on lymphoma growth in female mice as observed in male mice. We found that Letrozole treatment of C57BL6 female mice allografted with EG7 lymphoma cells caused a significant, although a slightly less robust stimulation of lymphoma growth compared to previously observed stimulation in male mice (Figure 4A). However, this effect was not seen in separate experiments when female sex hormones were removed by surgical ovariectomy (OVX) (Figure 4B), supporting that the stimulatory effect involves inhibition of estrogen synthesis.

## DISCUSSION

In this paper, we studied the underlying endocrine mechanisms that could explain a higher incidence and poorer prognosis in males *vs.* females as seen in several lymphomas. We show that removal of sex hormones by surgical castration in male mice accelerates lymphoma growth. This could be explained by both a higher number of proliferating Ki67-positive cells and a lower number of apoptotic tumor cells, suggesting that the lack of sex hormones stimulates tumor promotion. Previously it has been shown that lymphoma grafts grow faster in male vs. female mice and that the difference disappears following OVX, suggesting an inhibitory role of estrogens on lymphoma progression [26]. This conclusion was substantiated by the ability of estrogens, particularly ERβ agonists, to inhibit lymphoma growth when administrated to ovariectomized female or intact male mice. Lymphocytes and lymphoma cells mainly express ERβ [14, 16, 26]. An inhibitory role of estrogens on lymphoma development is in line with several epidemiological data as outlined in the Introduction. However, whether androgens themselves regulate lymphoma progression is not known. This is despite reports demonstrating AR expression in both normal lymphocytes and lymphoma cells [27, 34].

Surgical castration of male mice is a valuable and simple way to study the effect of the absence of sex hormones. However, in this case both male and female sex hormones are removed as estrogens are synthesized by conversion of androgens to estrogens by the enzyme aromatase in both males and females. Thus, male castration cannot distinguish whether androgens have a direct effect on lymphoma growth or whether an androgen effect is mediated indirectly through androgen conversion into estrogens.

To separate between these possibilities, we treated intact mice with AI or the AR antagonist Bicalutamide. While inhibition of estrogen synthesis by AI significantly promoted lymphoma growth, an AR antagonist had no significant effect. Another potent AR agonist, DHT, not converted to estrogens by aromatase, had no effect on lymphoma growth. Taken together, the results suggest that it is estrogens formed from androgens rather than androgens *per se* that inhibit lymphoma progression. This conclusion was substantiated by very low AR expression observed in the EG7 lymphoma studied (Suppl. Figure 1). Furthermore, the dose of Letrozole (10 μg/mouse/day) used in our experiments is a widely used treatment regime in experimental animal studies and results in reduced growth of grafted estrogen-dependent breast cancer as well as a reduced uterus size, two classical effects of decreased estrogen levels [35]. In addition, the stimulatory effect on lymphoma growth was observed using three structurally different AI's operating through both reversible and irreversible inhibition of the aromatase enzyme, making it highly likely that the lymphoma promoting effect indeed is an effect of aromatase inhibition. The promoted lymphoma growth was not due to reduced ERβ expression as its expression in the lymphoma tumor was not significantly altered by AI treatment (Suppl. Figure 2).

Importantly, the promoting effect of AI on lymphoma growth was seen in both a murine T cell lymphoma (EG7) and a human B cell lymphoma (Granta-519) suggesting that aromatase inhibition promotes lymphoma growth of both human and murine lymphomas as well as both T and B cell lymphomas, thus proposing a more general effect on lymphomas. The results also emphasize a general role of estrogens in inhibiting lymphoma pathogenesis and progression. Furthermore, a role of the immune system could be excluded because similar results were obtained in immune deficient NSG mice and in normal C57BL6 mice. Since AIs are used in the clinic as adjuvant therapy for female patients with hormone sensitive breast cancer [33], we also analyzed the effect of aromatase inhibition in female mice. In a similar way as in male mice, treatment with AI accelerated EG7 lymphoma growth in intact female mice. However, this effect was not observed after OVX. This can be explained by the fact that OVX not only abolishes the synthesis of estrogens but also the androgens, since the ovaries are the main source of androgens in female mice [36]. This is in contrast to human females where the adrenal cortex is the main source of androgens [37].

The conversion of androgens to estrogens in the mice most likely occurred in peripheral tissues known to express aromatase and not in the lymphoma tissue as analysis of aromatase expression was not detected in the EG7 tumors (data not shown). This is in contrast to some other endocrine-related malignancies e.g. breast cancer, which have been shown to express aromatase in the tumor tissue [38]. Further studies are required to analyze aromatase expression in clinical lymphoma material.

Our results raise a concern that treatment with AIs may increase the risk for lymphomas or at least accelerate lymphoma growth in cases were lymphoma initiation already has occurred. However, so far, according to our knowledge, no reports are available showing higher lymphoma incidence in AI-treated breast cancer patients. This may be explained by that AIs are relatively young drugs (introduced into the clinic in the beginning of the century) with so far to short follow up time from their start of use (less than 10 years) for such a side effect to be detected. Considering that lymphomas only constitute 4-5% of all cancers together with the limited follow up-period, very large breast cancer patient cohorts would be needed. An additional possibility is that *the* lack of estrogens can only contribute to lymphoma progression but not to lymphoma initiation, thus making the number of relevant breast cancer cases too few. Epidemiological studies of lymphoma incidence in breast cancer patients is also complicated by the fact that females with breast cancers do show an increased risk for secondary cancers including lymphomas compared to aged-mapped healthy woman [39]. In this study no reference to breast cancer treatment was reported. Additionally, the time period (usually 5 years) during which breast cancer patients are treated with AIs could be too short to increase the lymphoma risk.

Taken together, our results demonstrate that blocking estrogen synthesis via inhibition of the aromatase pathways and not androgen signaling causes accelerated lymphoma growth. These results highlight a protective role of estrogens in lymphoma pathogenesis and progression. As a consequence of this, physiological or therapeutically induced reduction of estrogen synthesis could be considered as a lymphoma risk.

## MATERIALS AND METHODS

### Cell lines

The murine T cell lymphoma cells (EG7) [40] and human B cell Granta-519 mantle cell lymphoma cells [41] used in this study were kept and maintained in RPMI 1640 medium supplemented with 10% fetal bovine serum, 2 mM L-glutamine, 100 IU penicillin/mL, and 100μg/mL streptomycin at 37°C in 5% CO_2_.

### Mouse models

C57BL6 male and female mice (6-8 weeks of age) were purchased from Charles River. Non-obese diabetic severe combined immunodeficiency NOD/SCID IL2γ-null (NOD.Cg-*Prkdc^scid^ Il2rg*^tm1Wjl^/SzJ) mice (referred to as NSG mice) were initially from The Jackson Laboratory (Bar Harbor, Maine, USA) and bred at the Animal Facility of Karolinska Institutet (NOVUM, Huddinge, Sweden). All animal experiments were carried out according to guidelines of the Karolinska Institutet and all the study protocols were approved by the Local Animal Experimentation Committee. Mice were kept in specific pathogen free conditions in 12 hours light-dark cycles and on soy-free diet during the experimental period.

### Animal microsurgery

Mice were anaesthetized using intraperitoneal injections of 100 μg Midazolam (Roche Diagnostics), 1.5 μg Domitor (Orion Corporation) and 0.05 μg Fentanyl (Janssen-Cilag) in 100 μl of physiological NaCl solution per mouse. Castration [42], ovariectomy [26] or sham-operations were done according to standard protocols and as described. After the operations, mice were allowed to recover for a week before tumor cell grafting was made.

### Tumor grafting, *in vivo* injections and tumor growth measurement

Mice were injected subcutaneously in the right flank with 0.5×10^6^ EG7 cells per C57BL6 mouse or with 15×10^6^ Granta-519 cells per NGS mouse in 100 μl sterile PBS. After palpable tumors developed, treatments with Letrozole (10 μg/animal) (Sigma), Bicalutamide (100 mg/kg) (Sigma) Anastrazole (200 μg/animal) (Sigma) or Exemestane (250 μg/animal) (Sigma) given subcutaneously once a day was initiated. All the drugs were dissolved in 100% ethanol and further diluted in rapeseed oil as a vehicle. Tumor size were monitored every day with a caliper and tumor volume (TV) was calculated according to the following formula: TV (mm^3^) = 0.5 × length (mm) × width^2^ (mm^2^).

### Fluorescence microscopy and image analysis of tumor cell proliferation and apoptosis

Tumor tissue was fixed overnight in cold 4% PFA in PBS, the following day washed with 50% ethanol and saved in 70% ethanol until sectioning and paraffin embedding. 5 μm thick slides were cut from the tumor tissue and mounted on glass slides. Antigen retrieval was done using Antigen unmasking solution (Vector Laboratories). The sections were permeabilized using 0.1% Triton X-100 in PBS after which they were incubated with primary Ki67 antibody (Novus Biologicals), followed by incubation with secondary anti-rabbit Cy3 antibody (Jackson Immunoresearch Ltd.), in a 0.1% BSA, 0.1% Triton X-100 PBS solution. Zeiss Axioplan2 fluorescence microscope was used for image acquisition. TUNEL-labelling was performed according to the manufacturer's instructions using an “In Situ cell death detection kit” from Sigma. Nuclei were counterstained with DAPI (Sigma). Images were obtained from 4 different non-overlapping fields and Ki67-positive and TUNEL-positive cells were counted using ImageJ (imagej.nih.gov/ij/) and a cell counter plugin.

### RNA isolation, cDNA preparation, qPCR

RNA was isolated from the tumor tissue using Qiazol reagent and RNAeasy Mini Kit (Qiagen). For tumor tissue disruption, a Qiashredder instrument was used. cDNA was prepared using a Reverted H Minus First Strand cDNA synthesis kit (Thermo Scientific) and oligodT method. qPCRs were run on ABIPrism 7500 instrument (Applied Biosystems) using duplicates, melting curve analysis and gene expression normalization was calculated using the ddCt method. Primer sequences used are given in Suppl Figure legends.

### Statistical analysis and data presentation

Statistical analysis was performed using unpaired, two-tailed t-test and p<0.05 was considered statistically significant using Microsoft Excel or GraphPad Prism 5.0. Means +/− SD values are presented. Significance between various groups is indicated with the following symbols (*, ¶, # p<0.05, **, ## p<0.01, *** p<0.001).

## SUPPLEMENTARY FIGURES



## Abbreviations

AI: aromatase inhibitor
AR: androgen receptor
DHT: dihydrotestosterone
ER: estrogen receptor
NHL: non-Hodgkin lymphoma
NSG: NOD/SCID IL2gamma-null mice
OVX: ovariectomy.
