# Supplementary material for: Inhibition of estrogen biosynthesis enhances lymphoma growth in mice
Source: Oncotarget. 2016 Mar 2;7(15):20718–27. doi: 10.18632/oncotarget.7843 (PMC4991487; doi:10.18632/oncotarget.7843)
Supplement: Supplementary file 1 [file oncotarget-07-20718-s001.pdf]

SUPPLEMENTARY FIGURES

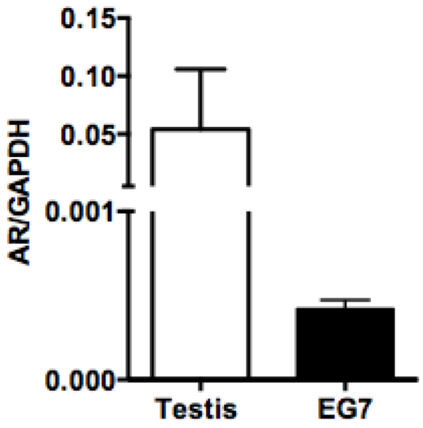

Supplementary Figure S1: Bars show gene expression of the androgen receptor normalized to GAPDH mRNA in mouse testicles isolated from C57BL6 male mice and EG7 tumor samples from the same mice. Bars are showing mean +/- SD. (n=3).

Primers used:
AR forward: AGAATCCCACATCCTGCTCAA
reverse: AAGTCCACGCTCACCATATGG
GAPDH forward: CTCGTCCCGTAGACAAAATGG
reverse: TGACCAGGCGCCCAATA

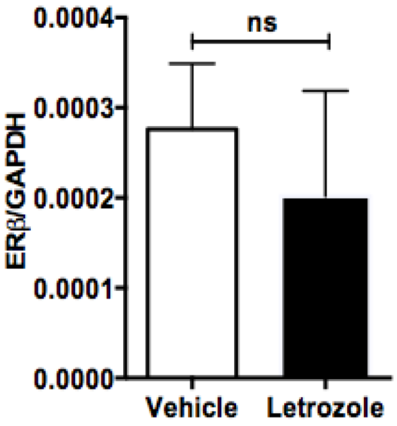

Supplementary Figure S2: Bars show the normalized gene expression of ERβ to GAPDH mRNA in EG7 tumor samples removed from vehicle and Letrozole treated mice. Bars are showing mean +/- SD. (n=5) ns=not significant.

Primers used:

ERβ forward: GCCAACCTCCTGATGCTTCT

reverse: TCGTACACCGGGACCACAT

GAPDH (see Suppl. Figure 1 legend)
